# Alveolar Dental Periostitis

**Published:** 1864-10

**Authors:** Henry S. Chase

**Affiliations:** Independence, Iowa


### DENTAL REGISTER OF THE WEST.

VOL. XVIII.] OCTOBER, 1864. [No. 10.

### Original Essays and Communications.

### ALVEOLAR DENTAL PERIOSTITIS.

Read before the Iowa State Dental Society.

BY HENRY S. CHASE, M. D.

The subject which has been assigned to me for an "Essay," has, during my whole professional life been the *opprobrium* of Dental Surgery.

It has taxed the minds of our best men for the last quarter of a century. Patient experiment and unflagging research are at last beginning to remove the cloud; and we behold the dawn of better days.

Inflammation of the alveolar dental membrane, is of frequent occurrence; indeed, hardly a day passes that we are not called upon to treat it. The causes which produce it, are various. The majority of cases result from death of the dental pulp. Many result from the constitutional effects of mercury; violent concussion of the teeth is another cause. It is frequently produced by suppressed perspiration, commonly called "taking cold." The disease is sometimes active, running to its termination in a very short time; at other times slow and insidious, taking weeks to produce pus formation. As a result of pulp-death, it is generally very rapid and severe. The symptoms are, in the incipient stage, a dull and slight soreness on occlusion of the jaws, gradually increasing to severe pain, throbbing in the offending part, and darting pains proceeding to the other teeth, and often to the eye and ear on the affected side. Severe symptoms, not accounted for by the non-professional, are often produced in other parts of the body, causing great anxiety to the patient and friends.

Through reflex action, the pneumogastric nerve is affected, and severe cramps are felt in the stomach, sometimes with nausea and vomiting. Cholera is not unfrequently a result of this disease; also tonic spasm of the masseter muscle; and paralysis of an arm or leg. The salivary glands are generally stimulated to excessive action, and pour out great quantities of their peculiar secretion; and their inflammation is not an unfrequent result of their sympathy.

If the disease runs its course unchecked by art, it usually results in abscess; though sometimes in resolution. The formation of pus is very unfortunate, as the symptoms are more severe, and, if unattended to, the results more grave.

The presence of pus in the socket of the tooth is known by increased soreness on percussion, and protrusion of the organ from its socket; attended by swelling of the gums and adjoining tissues,

Pus and an abscess are almost sure to be formed, if the disease is caused by death of the pulp, unless prevented by remedial means.

As the upper bicuspids often penetrate the antrum, we frequently have inflammation of that cavity as a result of *simple* periostitis, but *more* frequently of the aggravated form caused by extravasation of pus into it. In about thirty-six hours after pus is formed in the socket, fluctuation will generally be observed in the tumor it has caused, indicating its presence, which a sharp lancet will reveal. The pus usually makes its exit by absorption of the alveolus immediately opposite the end of the dentinal roots on the buccal surface of the jaws. Sometimes it takes place on the lingual side; at others it makes its way through the cancellated structure, and burrows for a long distance from the original seat of disease, spreading destruction in its track. At other times, though rarely, the absorbents attempt to carry away the offending matter, giving rise to frightful swelling of the whole face.

THE TREATMENT

Of this dreaded disease must be on general principles. The remedies must vary according to the cause, and stage of the disease. Many failures result from procrastination on the part of the patient. These cases are too often not presented to our notice until after pus formation, and then it is too often the case that the patient refuses any other treatment than extraction. The prognosis is favorable if under the following circumstances, viz: Simple periostitis; a good constitution; general good health; if caused by concussion; engorgement of capillaries from "taking cold." It is less favorable if caused by death of pulp, constitutional venereal taint, mercurial or strumous diathesis.

In regard to teeth as *classes* subject to this disease, those more or less successfully treated are mentioned in their order: Incisors, and cuspidati; bicuspids; upper molars; lower molars.

I had almost forgotten to observe that dental decay, even without exposure and death of pulp, is a frequent cause of periostitis. Without exposure to the air the pulp sometimes becomes inflamed, and transmits its condition to the alveolar dental membrane. In this case, after application of creosote to the cavity of decay, if the inflammation should not be reduced in three days, I should destroy the "pulp," and remove it.

I will illustrate my treatment by a few cases, occurring at intervals for the last twenty years. The treatment, which in these cases was successful, has, in other cases, not detailed, sometimes been *un*successful.

Case 1. July 1843. Mr. H., bilious temperament, called and wished for relief. Has had pain and soreness in right lower molars and bicuspids several weeks. They are slightly loose, and the gums engorged with blood. Was mercuricalized some years since. Health rather poor; liver inactive.

Lanced the gums. To take one grain iodide potass., thrice daily. To have generous diet. Reported second week better. To continue medicine. Third week cured.

CASE 2. March, 1845. Mr. F. sent for me: found the patient feverish, and suffering with great pain in lower incisors, which were slightly loose and sensitive to touch.

Lanced the gums. To have hot foot bath. Take mercury, five grains to night: in the morning dose sulph. magnesia. Called next day and found him better: the following day well.

CASE 3. May 1845. Miss B. called to have right upper central incisor *extracted*, being very painful. It had been filled one month previously, nerve not nearly exposed. A slight discoloration told me that the nerve was dead. With a very fine drill I pierced the gum just above its margin and entered the pulp cavity. On withdrawing the instrument it was followed by a rush of purulent matter. Immediate relief was experienced, and no after trouble known for many years.

CASE 4. Mr. C. has had severe throbbbing pain in left inferior molar, several days. Was filled six months ago; at which time pulp was destroyed: drilled to pulp cavity and syringed with alcohol and creosote. Cured.

CASE 5. Mr. G., age 25. Called to have right upper molar extracted: he said it had ached three days and he could stand it no longer. Found the tooth perfectly sound, and also those adjoining. Refused to extract. Patient to take mercurius vivus; 3d dec. prep., once in two hours until better. Called in twenty-four hours and said the first three doses cured him. In this case the patient had a "common cold," and the lining membrane of the antrum was affected as was indicated by pain felt on pressure over this cavity. Doubtless the roots of the molar pierced this cavity, and thus participated in the inflammation. In this case a *want* of medical knowledge would have led me to have killed and removed the pulp. We can not lay too much stress on the acquirement of a thorough knowledge of MEDICAL SCIENCE by those who seek to enter the Dental Profession. The want of it will surely degrade the science to a mere trade.

CASE 6. July 5th. Mr. M., age 35. Applied to have the right upper lateral incisor extracted. Has a small plug in good condition on the lateral surface, which was placed there in 1857, over an exposed nerve. It was painful at times for about one year and then "ulcerated;" the discharge taking place on labial surface of gums, apparently about half way between the end of the root and alveolar border. Has continued since that time troublesome; and lately so painful as to determine the patient on *extraction.* I declined to extract the tooth, and recommended an operation, which I immediately commenced. Beginning with a round burr drill, I hollowed out the posterior portion of the central part of the inscior near the cutting edge, and carried the groove upwards until there was a shelf of bone sufficient to retain in place a drill No. 17 of the gauge plate. I now drilled straight into the pulp cavity, enlarging it, and going through the end of the root with a drill of No. 22 gauge plate. Syringed with alcohol, and wiped out with creosote, leaving considerable of the latter in the canal. Making a plug of soft rubber, I forced it tightly into the root as far as possible, and soon had the satisfaction of seeing the caustic effects of the creosote acting on the mucous membrane of the gum about the fistulous opening through which the purulent secretions had formerly discharged themselves. Dismissed the patient, who is to call again in a few days.

July 10th. Patient called, and reports no pain, but has a feeling of uneasiness. Repeated the injection of creosote, but was unable to make it show its effects on the gum as before: conclude that the fistula has healed up. Wiped canal dry and plugged with soft rubber; dismissing patient for two days.

July 12th. Tooth still remaining well, and uneasy feeling gone. I wiped with creosote and iodine, and plugged again with rubber, as I had no time to plug with metal to-day.

July 15th. Plugged canal with tin foil, to remain one year unless there should be a return of disease.

Aug. 6th. Tooth still continues perfectly well, and both myself and patient consider the cure complete.

CASE 7. I report this case, because it was a very bad one, and had an unfortunate termination.

Nov. 1863. Mr. F., age about 35, billious temperament, called to have left under molar extracted; but very much wished it could be saved. Applied arsenical paste for twenty-four hours and then removed pulp and treated with creosote one week. Syringed tooth with alcohol and plugged with "Wood's Alloy."

Jan. 1864. The tooth is sore and aches; drilled, but no exit of pus or other matter; forced a little creosote into the pulp cavity.

Feb. Patient sent for me. Had been sick about a month under his family physician, to whom he had applied to have his tooth extracted the day after his last visit to my office; it being Sunday and my office closed was his excuse for not applying to me. Physician declined to extract. High fever followed, with sore throat; cramps in the stomach; swelling of the parotid and sub-lingual glands of the affected side; excessive flow of saliva; followed by rigidity of the masseter muscles. Nourishment and medicines were now taken through an opening caused by loss of a central incisor. Pus, mixed with large quantities of saliva, was daily evacuated. Owing to absence from town, I was not called sooner. I found the patient somewhat better of the symptoms before mentioned. The jaws could be slightly opened, and I extracted the tooth which had originated this trouble: as it was for this that I had been called, and not for a general treatment of the case. However I ventured to recommend tinc. iodine as an application to socket of tooth, and an internal remedy of a similar character. The next week was sent for again to extract the second bicuspid which was sore and loose, and from its margin of gum was weeping pus continually. Found that my recommendation of remedies had not been followed in the least. Pus also continued to be evacuated from the socket of the molar before extracted. I had forgotten to say that the first and second molars of this side were missing.

I again warned patient that medical treatment for this disease should be instituted.

March 17th. Patient called at my office; general health much improved; but disease of the jaw still continued, and the first bicuspid was now involved in the matter, being sore, loose, and discharging pus around its alveolar border. Again insisted on medical treatment, to which patient acquiesced. Take iodide potassa first, dec. prep., thrice daily. Nourishing diet of eggs, oysters, ale. After one week found the patient much improved every way; still there was swelling of the face and a purulent discharge; but the inflammation was not extending, and the first bicuspid was saved. I wished now to make an inscision lengthways of the jaw, through the gums and periosteum as far as the disease had extended, for the purpose of evacuating any purulent matter that might be burrowing there, but patient refused In a few days, his general health improving, he went to New York to purchase goods, and while there had professional advice, and a sequestra of bone removed from his jaw. Since his return some other small pieces have been removed, as I am told, for he has lost confidence in my skill, and imputes to me the whole cause of his troubles. This is the most severe case I ever had, and was under my control very little.

In the Fifth Edition of Dr. Harris' work, page 468, a much severer case is reported; the patient loosing sixteen teeth in a few months.

When alveolar abscess has formed it should be opened at the most dependent portion, that the pus may all easily escape, and a little lint left in the cut, to keep it open a day or two longer; otherwise the incision might heal by first intention and the abscess fill again immediately.

In the cases reported we have seen that thorough cleansing of the root canals with alcohol, and injections of creosote and iodine, have been of great value, in permanently curing chronic inflammation of the alveolar periosteum. The way to reach the seat of disease in different cases must be left to the judgment of the practitioner. The most difficult cases I conceive to be the molars. If we could be sure of finding the *ends* of those roots, readily, through the gums and alveoli, doubtless it would be good practice to drill down to their end, inject with creosote and iodine.

It sometimes happens that alveolar abscess evacuates itself into the autrum, causing inflamation of that cavity, with distressing and protracted symptoms. Protracted, because it is so often mistaken for catarrh. In this case the autrum should be perforated between the roots of the second bicuspid and first molar teeth, unless an opportunity offers through the socket of a *worthless* tooth of this kind. The opening should be as large as a No. 12 drill. Syringing this cavity with a weak tinct. of iodine and opium will be found beneficial, repeated once in two or three days until better. Mercury as an *internal* remedy will be indicated.

I have thus, gentlemen, endeavored to give you *my experience* in the treatment of the disease under consideration; believing it unnecessary to detail the treatment recommended by authors, as the same sources of information are open to you as to myself.

*Independence, Iowa, August 9th,* 1864.

